# Hereditary cancer syndrome-associated pathogenic variants are common in patients with hematologic malignancies subsequent to primary solid cancer

**DOI:** 10.7150/jca.54169

**Published:** 2021-05-19

**Authors:** Joowon Oh, Yu Ri Kim, Yoonjung Kim, Boyeon Kim, Kyung Sun Park, Seong-Hyeuk Nam, Kyung-A Lee

**Affiliations:** 1 Department of Laboratory Medicine, Sheikh Khalifa Specialty Hospital, Ras Al Khaimah, United Arab Emirates.; 2 Division of hematology, Department of internal medicine, Yonsei University College of Medicine, Seoul, Korea.; 3 Department of Laboratory Medicine, Yonsei University College of Medicine, Seoul, Korea.; 4 Department of Laboratory Medicine, School of Medicine, Kyung Hee University, Seoul, Korea.; 5 SD Genomics Co., Ltd., Seoul, Korea.

**Keywords:** hematologic malignancy, germline predisposition to cancer, clonal hematopoiesis of indeterminate potential, next-generation sequencing, therapy-related myeloid neoplasms

## Abstract

**Background:** As the number of long-term survivors of solid cancers keeps increasing, risk assessment of secondary hematologic malignancies is important for the prognosis of the patient. Germline genetic predisposition to secondary hematologic malignancy has been studied widely in myeloid neoplasms and rarely in lymphoid neoplasms. This study aimed to profile the mutational spectrums of patients with subsequent lymphoid tissue neoplasm to shed some light on the understudied area.

**Methods:** In total, 39 patients who had primary solid cancer and subsequent hematologic malignancies were enrolled. We performed two next-generation sequencing (NGS) panel tests encompassing hereditary cancer predisposition genes and genes related to clonal hematopoiesis of indeterminate potential (CHIP). All statistical analyses were performed using R 3.5.1.

**Results:** We found 8 of 39 patients with germline mutations in cancer predisposition genes; 4 of 18 patients had therapy-related myeloid neoplasms (22.2%); and 4 of 15 patients had secondary lymphoid malignancies (26.7%). Notably, of 14 patients who initially suffered from thyroid cancer, 5 patients (35.7%) had germline mutations. Malignancy of lymphoid tissue showed no association with radioactive iodine therapy but was observed to a greater extent in germline mutation-positive thyroid cancer patients regardless of their history of treatment. We observed that 24 of 39 patients (61.5%) were CHIP carriers. Patients who had secondary lymphoid malignancy were less likely to have CHIP than those who had myeloid malignancy.

**Conclusions:** In patients with primary solid cancer who are planning to undergo cytotoxic chemotherapy, radiotherapy, or radioactive iodine therapy, an initial assessment with germline mutation testing using an expanded NGS panel, including low, moderate, and high-risk cancer-associated genes, and somatic CHIP mutation testing can screen the patients who are at risk of developing therapy-related myeloid and lymphoid malignancies. Through careful screening and monitoring throughout the treatment process, patients can benefit from the early detection of secondary malignancies and receive proper treatment.

## Introduction

Therapy-related hematologic malignancies occur in patients exposed to cytotoxic chemotherapy and/or radiation therapy. Studies on the genetic spectrums of therapy-related myeloid neoplasms (t-MNs) have revealed several associated genetic abnormalities that can be categorized as follows: i) chromosomal abnormalities in hematopoietic stem cells 1, ii) genetic mutations in tumor suppressor genes, oncogenes, genes that regulate cell proliferation or DNA methylations such as *TP53*, *TET2*, *DNMT3A*, and *RUNX1*, and *RAS* family genes 2, 3, iii) clonal hematopoiesis of indeterminate potential (CHIP) among hematopoietic stem cells 4-6, and iv) inherited mutations in cancer predisposition genes 7. While identifying somatic abnormalities in malignant cells is essential for predicting the prognosis, relapse, and survival of the patients 8, 9, testing for inherited mutations in cancer predisposition genes is important for risk assessment in some patient groups, such as patients with a familial history of cancer or cancer patients who are recommended to undergo cytotoxic chemotherapy or radiation therapy.

In the studies concerning germline genetic predisposition to hematologic malignancy, inherited mutations in high-penetrance cancer predisposition genes have been identified and well described 7. Moderate or low-penetrance genes have been recently studied, where some cases indicated that inherited mutations in rather low-risk genes can be associated with susceptibility to t-MN 2. For instance, *WRN*-mutated Werner's syndrome patients have a 10% incidence of developing cancer and increased risk of acute leukemia 10. Moreover, therapy-related myelodysplastic syndrome (MDS) has been reported in patients with Werner's syndrome along with thyroid cancer who were exposed to radioactive iodine therapy 11. While the genetic spectrum of t-MN has been widely studied and reviewed 3, 12, the genetic study of therapy-related lymphoid malignancy has rarely been carried out. As long-term survivors of solid cancers keep increasing, secondary lymphoid malignancies, which arise in relatively the latter part of the disease-free period 13, should be recognized, and their risk factors should be assessed. A recent study of lymphoid malignancy patients with familial traits showed 52% comorbidities with multiple types of solid cancers and found several pathogenic mutations in cancer predisposition genes 14. In this regard, we hypothesized that an increased risk of secondary lymphoid malignancies after chemotherapy or radiation therapy can be explained by germline mutations in cancer predisposition genes. Hence, in the current study, we enrolled patients with hematologic malignancies, either from myeloid or lymphoid origins, who previously had solid tumors and tested them for germline mutations using an expanded next-generation sequencing (NGS) panel that included low-, moderate-, and high-risk cancer-associated genes. Considering the paucity of genetic studies concerning germline predisposition to secondary lymphoid malignancy, profiling the mutational spectrums of subsequent lymphoid tissue neoplasm patients in this study could provide novel insights for the better management of cancer patients.

## Methods

The study subjects were enrolled retrospectively. We reviewed the bone marrow aspiration reports of 852 patients from January 2013 to April 2018 at one university hospital. Via screening, 46 patients diagnosed with myeloid or lymphoid malignancies with previous histories of solid tumors were selected. Finally, 39 patients were enrolled for analyses, after excluding patients with insufficient preserved bone marrow samples at research specimen banking. All samples used in the analysis of CHIP were collected at the diagnosis with subsequent hematologic malignancies. To distinguish germline mutation from somatic mutation, we used follow-up bone marrow samples at the time of complete remission or peripheral blood samples with low or no blast observed. This study was approved by the Gangnam Severance Hospital Institutional Review Board (IRB approval number: 2018-0316-01). Detailed information on the targeted NGS panel sequencing and statistical analyses are described in Supplementary Document 1 and Supplementary Tables 1-2.

## Results

The characteristics of the patients are described in Table 1. The median age of the participants at the time of initial cancer diagnosis was 61 years (Q1Q3; 51.0, 68.5), and the median follow-up time was 7 months (Q1Q3; 3.5, 36.0). The most common primary solid cancer was thyroid cancer (35.9%, 14/39), followed by breast cancer (12.8%, 5/39) and colorectal cancer (10.3%, 4/39). Twenty-four patients had a subsequent hematologic malignancy after being diagnosed with the primary cancer; acute myeloid leukemia (AML) in 7 cases, MDS in 11 cases, and myeloproliferative neoplasm (MPN) in 6 cases. Fifteen patients had secondary malignancy in lymphoid tissue; eight patients had diffuse large B-cell lymphoma (DLBCL), five patients had plasma cell myeloma (PCM), and two patients had chronic lymphocytic leukemia (Supplementary Table 3). Twenty-six patients (66.7%) received chemotherapy or radiotherapy as treatment for the initial solid cancer. Of 12 patients who had undergone chemotherapy, 4 were treated with alkylating agents and 2 with topoisomerase II inhibitors (Supplementary Table 3).

**Figure 1:**
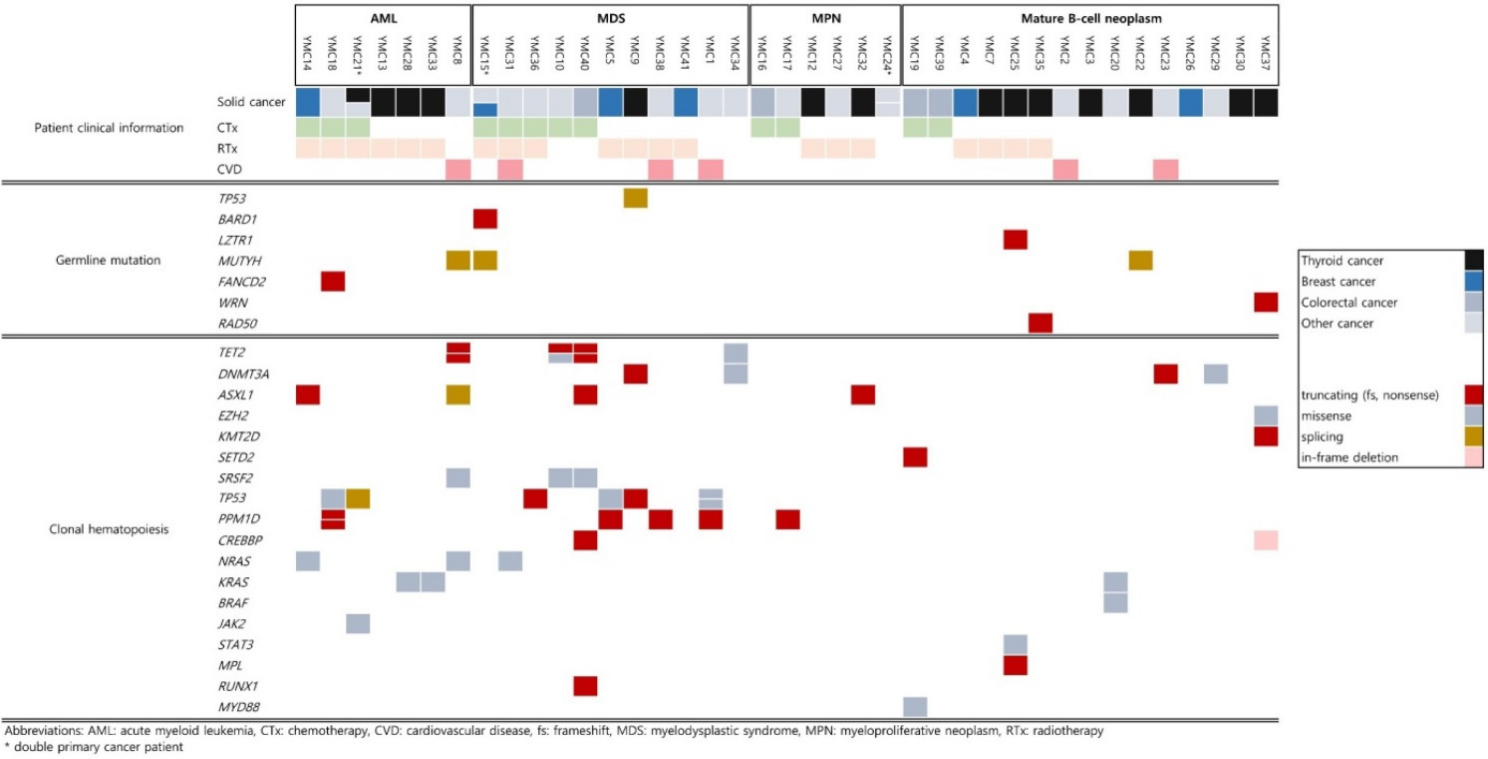
Summary of germline mutations and clonal hematopoiesis of indeterminate potential (CHIP) mutations. These mutations were observed in 39 cancer patients according to the subsequent hematologic or lymphoid tissue malignancies.

Detailed mutational findings of all participants are described in Figure 1. In total, 8 out of 39 patients had germline mutations in cancer predisposition genes [4 of 18 patients with t-MN (22.2%) and 4 of 15 patients with secondary lymphoid malignancy (26.7%)]. Besides the pathogenic variants in the *TP53* gene, responsible for the Li-Fraumeni Syndrome, the rest of the pathogenic mutations that were found in *BARD1*, *LZTR1*, *MUTYH*, *FANCD2*, *WRN*, and *RAD50* are moderate-risk alleles. One patient who suffered from ovarian cancer and breast cancer with therapy-related MDS (t-MDS) had germline mutations in *BARD1* and *MUTYH*. The patient was first diagnosed with ovarian cancer at the age of 75, which is compatible with the moderate penetrance that the putative disease-causing gene has. Karyotyping of bone marrow when diagnosed with t-MDS showed monosomy 5 and monosomy 7, explained by the use of chemotherapy with alkylating agents and radiotherapy. The monoallelic (heterozygous) *MUTYH* mutation (NM_001128425.1:c.934-2A>G) found in the patient above was also detected in two other patients in this study group. Three patients who had the same *MUTYH* splicing mutation were first diagnosed with ovarian cancer, thymoma, and thyroid cancer, respectively. Two patients with thymoma and thyroid cancer underwent only surgical resection of the tumor. They had subsequent MDS and DLBCL. One patient out of the 18 t-MN patients (5.6%) had a germline mutation in the *FANCD2* gene (NM_033084.3: c.757C>T, p.Arg253*). A nonsense germline mutation in *RAD50* (NM_005732.3: c.1106C>G, p.Ser369*) was observed in one patient in our study. The patient was first diagnosed with thyroid cancer; after surgical removal, the patient underwent radioactive iodine therapy (RAIT) and was diagnosed with PCM 68 months later.

Notably, out of the 14 patients who initially suffered from thyroid cancer, 5 patients (35.7%) had germline mutations. Malignancy of lymphoid tissue showed no association with RAIT but was observed to a greater extent in germline mutation-positive thyroid cancer patients regardless of their RAIT status (Figure 1). The results of our study show that RAIT is associated with secondary myeloid malignancies with borderline statistical significance (*P* value = 0.076, Table 2). Unlike the previous study with CHIP analysis where the average age at the initial diagnosis of thyroid cancer was around 64 15, the average age in our thyroid patient cancer group was around 50.

Of 39 patients, 24 (61.5%) were CHIP carriers. The median age of the CHIP-positive and CHIP-negative patients was 62.5 years (Q1Q3; 52.0, 71.5) and 58.0 years (Q1Q3; 50.5, 64.5), respectively. The median follow-up time was 6 months (Q1Q3; 2.0, 9.5) for the CHIP-positive patients and 22.5 months (Q1Q3; 7.5, 41.5) for the CHIP-negative patients. Patients who had secondary lymphoid malignancy were less likely to have CHIP than those who had myeloid malignancy. Seventy-five percent of the patients (18 out of 24) with subsequent myeloid malignancies had CHIP mutations, which is similar to the previous case-control studies where 62-71% of the t-MN cases had CHIP mutations 5, 6. As suggested in literature, CHIP caused by cytotoxic therapy is associated with high frequencies of mutations in *TP53* and *PPM1D* 16, 17. Consistent with this, in our study, the two most frequently mutated genes in the CHIP-positive cases were *TP53* (7/24, 29.2%) and *PPM1D* (5/24, 20.8%) in 7 patients who underwent either cytotoxic chemotherapy or radiation therapy. Although statistically significant differences were not observed between the CHIP-positive and CHIP-negative groups with regard to the incidence of cardiovascular disease (CVD) owing to the small number of cases, out of 6 patients who had CVD, 5 cases had CHIP mutations. The CHIP mutations in the CVD patients included genes such as *TET2*, *ASXL1*, *DNMT3A*, and *TP53*.

## Discussion

Although the positive rates of pathogenic variants are different among the types of cancer and the extent of the NGS panel, they are generally considered to be around 7.4%~12.6% when using the core gene panel and up to 20% when using an extended panel with all known cancer susceptibility genes^18^-^21^. The results from our study are consistent with previous studies in that pathogenic mutations in low to moderate penetrance genes are present in t-MN patients. We should note that regardless of the mode of inheritance, there are previous studies which correlates monoallelic mutation and cancer susceptibility (Table 3). A recent study investigated the correlation of 16 Fanconi anemia (FA)-related genes with myeloid neoplasms, including both de novo AML and t-MN 22. The authors suggested that heterozygous carriers of FA variants may have increased susceptibility to environmental carcinogens and to the DNA-damaging action of cytotoxic therapy used to treat primary tumors, leading to de novo or secondary leukemogenesis 22. In the current study, 1 patient out of 18 t-MN patients (5.6%) had a germline mutation in the *FANCD2* gene (NM_033084.3: c.757C>T, p.Arg253*). The mutation was reported as 'pathogenic' in FA patients with a severe phenotype and has been associated with an increased risk of intraductal papillary mucinous neoplasms 23. The FANCD2 protein plays a key role in the initiation of the FA pathway, a DNA damage repair mechanism. FANCD2 deficiency can lead to uncontrolled cell proliferation, leading to FA-associated malignancies, and therefore increase cancer susceptibility sporadically in the general population 24, 25.

The monoallelic (heterozygous) *MUTYH* mutation (NM_001128425.1:c.934-2A>G) was found in three patients in this study group. According to the gnomAD Exomes database (https://gnomad.broadinstitute.org/), this specific splicing mutation has a minor allele frequency of 1.5% in the East Asian population, which is lower than the incidence rate observed in our study group (7.7%, 3/39). Biallelic and monoallelic *MUTYH* mutations are known to be associated with increased risk of colorectal cancer (CRC) 26, 30 as well as gastric cancer, hepatobiliary cancer, endometrial cancer, and breast cancer 31. Out of 15 patients, 4 (26.6%) with subsequent mature B-cell neoplasms had germline pathogenic mutations in cancer susceptibility genes; *MUTYH* and *WRN* in 2 subsequent DLBCL patients and *RAD50* and *LZTR1* in 2 subsequent PCM patients. Besides *RAD50*, which is suggested to have an influence on susceptibility to DLBCL 32, *MUTYH*, *WRN*, and *LZTR1* have never been reported to have associations with lymphoid malignancies. We showed that monoallelic mutations in the genes involved in the DNA damage response pathways might be associated with various types of cancer, including both de novo and therapy-related hematologic malignancies, either from myeloid or lymphoid origins. The high prevalence of mutations in cancer susceptibility genes in our study group indicates the importance of genetic testing in patients with primary solid cancers for the risk assessment of secondary hematologic malignancies. Indeed, a recent study involving long-term childhood cancer survivors showed significantly increased rates of subsequent neoplasms among carriers of pathogenic mutations in DNA repair genes 33.

An increased risk of a second primary malignancy after RAIT was proposed in several studies 34-36, and a recent analysis suggested that RAIT is associated with an increased risk of AML and chronic myeloid leukemia but not of malignancies of the lymphoid tissue or of PCM 37. Our study results also indicate that RAIT is associated with secondary myeloid malignancies. Given the fact that age is the most relevant factor for CHIP existence 38, 39, our result that six out of ten RAIT-treated patients (60%), whose average age was 50, showing CHIP positivity suggests that CHIP is associated with RAIT. This reinforces the result from the previous study 15, which was limited by an older-aged cohort composition. Although the CHIP analysis in our study was performed at the diagnosis of the second malignancy, the current understanding of the multistep pathogenesis of cancer suggests that individuals with clonal mutations may have already had smaller clones of existing mutations and they were in the path toward the evolution of the malignancy 40. In this context, thyroid cancer patients who are candidates for adjuvant RAIT could benefit from molecular testing of the hereditary cancer NGS panel before treatment. Furthermore, monitoring the CHIP composition before and after treatment could help with the early detection of therapy-related hematologic malignancies. Upon further confirmation of this suggestion by well-designed studies, more patients will benefit from avoiding unnecessary adjuvant RAIT, leading to improved overall survival.

Reports on the association between CHIP and chemotherapy-subsequent lymphoid malignancies are rare. While the risk of t-MN is known to be higher when lymphoma patients with CHIP undergo autologous stem cell transplant 41, the risk of the development of mature B-cell neoplasms in patients with CHIP who undergo chemotherapy or radiotherapy against primary solid cancers is not widely studied. In our study, 40% (6 out of 15) of the patients with subsequent mature B-cell neoplasms had CHIP mutations. Unlike t-MN patients with CHIP most commonly involving *TP53* and *PPM1D*, a variety of genes were observed in the patients with lymphoid tissue malignancies: *DNMT3A*, a DNA methyltransferase; *EXH2* and *KMT2D*, which are involved in histone modification; *CREBBP*, which is involved in the p53-dependent signal pathways; *KRAS* and *BRAF*, with roles in the RAS/MAPK pathway; and *STAT3*, which plays a role in cell proliferation. Further investigations with age-matched controls are needed to reveal the true associations between CHIP and therapy-related lymphoid tissue malignancies.

Our study is limited by small number of study population, retrospective sample collection and the lack of age-matched healthy control. Large-scaled prospective study in cancer patient with concurrent or subsequent hematologic malignancies should be done to add statistical and clinical robustness to the current study.

## Conclusions

In patients with primary solid cancer who are planning to undergo cytotoxic chemotherapy, radiotherapy, or RAIT, initial assessment with germline mutation testing using an expanded NGS panel comprising low-, moderate-, and high-risk cancer-associated genes and somatic CHIP mutation testing can give clue to the patients who are at risk of developing therapy-related myeloid and lymphoid malignancies. Through careful screening and monitoring throughout the treatment process, patients may benefit from the early detection of secondary malignancies and receive proper treatment.

## Supplementary Material

Supplementary materials.

Supplementary tables.

## Figures and Tables

**Table 1:** Patient characteristics of the study population

	All (N=39)	germline positive (N=8)	germline negative (N=31)	*P* value
**Age (year)**				
Median [interquartile range]	61 [51.0;68.5]	63.5[57.0;68.5]	58.0[50.0;68.5]	0.338
**Sex**				
Female	21 (53.8%)	6 (75.0%)	15 (48.4%)	0.343
Male	18 (46.2%)	2 (25.0%)	16 (51.6%)	
**Type of primary solid tumor**				
Thyroid cancer	14 (35.9%)	5 (62.5%)	9 (29.0%)	0.227^a^
Breast cancer	5 (12.8%)	0 (0.0%)	5 (16.1%)	
Colorectal cancer	4 (10.3%)	0 (0.0%)	4 (12.9%)	
Other*	16 (41.0%)	3 (37.5%)	13 (41.9%)	
**Secondary malignancy**				
Hematopoietic	24 (61.5%)	4 (50.0%)	20 (64.5%)	0.73
Lymphoid	15 (38.5%)	4 (50.0%)	11 (35.5%)	
**Type of hematologic malignancy**				
AML	7 (17.9%)	2 (25.0%)	5 (16.1%)	0.53
MDS	11 (28.2%)	2 (25.0%)	9 (29.0%)	
MPN	6 (15.4%)	0 (0.0%)	6 (19.4%)	
Mature B-cell neoplasm	15 (38.5%)	4 (50.0%)	11 (35.5%)	
**Treatment^b^**				
Radiotherapy	20 (51.3%)	5 (62.5%)	15 (48.4%)	0.753
Chemotherapy	12 (30.8%)	2 (25.0%)	10 (32.3%)	1.000
**Disease-free survival (months)**	58 [29.0; 77.0]	51.4 ± 25.1	60.5 ± 44.9	0.585
**Comorbid cardiovascular disease**	6 (15.4%)	1 (12.5%)	5 (16.1%)	1.000
**Overall survival (months)**	7.0 [3.5;36.0]	8.0 [ 1.0;46.0]	7.0 [ 4.5;29.0]	0.958
**Death**	9 (23.1%)	9 (23.1%)	7 (22.6%)	1.000

*: ovarian cancer, prostate cancer, lung cancer, common bile duct cancer, thymoma, hepatic cell carcinoma, gastric cancer, bladder cancer, esophageal cancer. ^a^ *P* values represent a comparison of germline-positive/germline negative across all types of primary solid tumor categories. ^b^ The total number of patients with radiotherapy or chemotherapy is 26. Six patients had both radiotherapy and chemotherapy. Abbreviations: AML: acute myeloid leukemia, MDS: myelodysplastic syndrome, MPN: myeloproliferative neoplasm.

**Table 2:** Patients' demographic data in the primary thyroid cancer group (n=14)

RAIT (n=10)	Surgery only (n=4)	*P* value
**Female (n)**		
7	4	0.607
**Age at diagnosis (mean ± SD)**		
47.9 ± 11.9	53.5 ± 9.5	0.420
**Age at secondary malignancies (mean ± SD)**		
52.1 ± 12.0	59.0 ± 9.6	0.329
**Myeloid malignancy**		
7	0	0.076
**Type of secondary malignancies**		
AML (4)	DLBCL (3)	
MDS (1)	PCM (1)	
CML (2)		
DLBCL (1)		
PCM (2)		
**CHIP positive (n)**		
6	1	0.554
**Germline positive (n)**		
3	2	0.930
**Survival (months)**		
32.0 ± 24.0	15.5 ± 15.6	0.233

Abbreviations: AML: acute myeloid leukemia, CHIP: clonal hematopoiesis of indeterminate potential, CML: chronic myelogenous leukemia, DLBCL: diffuse large B-cell lymphoma, MDS: myelodysplastic syndrome, PCM: plasma cell myeloma, RAIT: radioactive iodine therapy, SD: standard deviation.

**Table 3:** Germline pathogenic mutation and studies on cancer susceptibility

Confirmed germline variants: Gene, variants, zygosity	Interpretation of Variant by ACMG guideline^a^	Population frequency^a^	# case in this study	OR (95% CI), *P* value	Mode of Inheritance^b^	Studies on monoallelic variant and its cancer susceptibility
*MUTYH*, NM_001128425.1:c.934-2A>G, heterozygous	Pathogenic: PVS1, PP3	0.0152	3	5.39 (1.65-17.61), P = 0.0053	AR	E Theodoratou et al, 2010: Monoallelic MUTYH mutations are associated with increased risk of colorectal cancer 26
*TP53*,NM_000546.5:c.994-1G>C, heterozygous	Pathogenic: PVS1, PM2, PP3, PP5	0	1	-	AD	*
*BARD1*, NM_000465.3:c.448C>T, p.(Arg150*), heterozygous	Pathogenic: PVS1, PM2, PP3, PP5	0	1	-	AD	*
*FANCD2*, NM_033084.3:c.757C>T, p.(Arg253*), heterozygous	Pathogenic: PVS1, PM2, PP3, PP5	0	1	-	AR	E Barroso et al, 2006: The data indicate that a relationship between *FANCD2* and sporadic breast cancer risk may exist 25
*LZTR1*,NM_006767.3:c.27dup, p.(Gln10Alafs*830), heterozygous	Pathogenic: PVS1, PM2, PP3, PP5	0.000336	1	-	AD, AR	N/A
*RAD50*, NM_005732.3:c.1106C>G, p.(Ser369*), heterozygous	Pathogenic: PVS1, PM2, PP3, PP5	0	1	-	Not known	K Heikkinen et al, 2006: There's and effect for *RAD50* haploinsufficiency on genemic integrity and susceptibility to cancer 27
*WRN*,NM_000553.4:c.968C>G, p.(Ser323*), heterozygous	Pathogenic: PVS1, PM2, PP3	0	1	-	AR	S Ding et al, 2007: There is tumorigenic contribution of *WRN* to breast cancer development 28. M Wirtenberger et al, 2006: *WRN* act as low-penetrance familial breast cancer susceptibility genes 29.

^a^ Standards and Guidelines for the Interpretation of Sequence Variants: A Joint Consensus Recommendation of the American College of Medical Genetics and Genomics and the Association for Molecular Pathology. ^b^ Population frequency based on The Genome Aggregation Database (gnomAD) Exome database, East Asian population. Abbreviations: AD: autosomal dominant, AR: autosomal recessive, CI: confidence interval, N/A: not available, OR: odds ratio. *Autosomal dominant cancer susceptibility gene.
