# Supplementary material for: Hereditary cancer syndrome-associated pathogenic variants are common in patients with hematologic malignancies subsequent to primary solid cancer
Source: J Cancer. 2021 May 19;12(14):4288–94. doi: 10.7150/jca.54169 (PMC8176409; doi:10.7150/jca.54169)
Supplement: Supplementary file 1 — Supplementary materials. [file jcav12p4288s1.pdf]

Methods

Targeted NGS panel sequencing

A customized panel using a hybridization capture-based next-generation sequencing assay was designed. This panel covered all protein-coding exons of 31 genes for a clonal hematopoietic of indeterminate potential (CHIP) analysis (Table S1) and 108 genes for a hereditary cancer analysis (Table S2). DNA was isolated on a Chemagic 360-D instrument (PerkinElmer, Baesweiler, Germany), based on magnetic bead technology, according to the manufacturer's instructions. Isolated genomic DNA was sheared on Qsonia Q800R2 (Newtown, CT, USA). DNA concentrations were measured using the Qubit 3.0 Fluorometer (Invitrogen, California, USA) and Qubit dsDNA HS Assay Kit (Invitrogen, California, USA). The KAPA library amplification kit was used to prepare bar-coded DNA molecules with library adapters. DNA fragments were captured using oligonucleotide probes. Sequencing was performed with 2 × 150 bp NextSeq runs (Illumina, San Diego, CA, USA). The NGS quality control matrix was at least 2,000× mean reads on target with a minimum of 500× coverage on >98% for the CHIP analysis and at least 200× mean reads on target with a minimum of 20× coverage on >99.9% target bases for the hereditary cancer analysis.

For the CHIP analysis, the sequencing reads were aligned to the human reference genome (hg19) with decoy sequences (hs37d5) using BWA-MEM (v0.7.15)[1]. Picard (v2.9.2) and GATK (v3.7.0)[2] were used for the removal of duplicated reads and recalibration of the base quality score, respectively. The single nucleotide variants (SNVs) and small insertions and deletions (INDELs) were called by MuTect2 (v3.7.0)[3] with default parameters. We filtered out the variants according to the following rules: (a) variants with a very low (< 0.01) variant allele frequency (VAF) except for the hotspot mutations in COSMIC database (https://cancer.sanger.ac.uk/cosmic) or null variants in a gene where loss of function is a main mechanism; (b) common variants with population allele frequency >1% in the gnomAD database[4]; and (c) other frequently detected variants that are likely to be technical artifacts. We

considered mutations repetitively reported in COSMIC or previous studies on CHIP or null variants in a gene where loss of function is a main mechanism with a VAF of at least 0.01 as candidate somatic mutations. In the case of serial samples, if there was a reliable variant in one sample, this variant in other samples from the same patient was not removed despite the fact that it had a very low VAF (< 0.01). For validation and quality control of the CHIP NGS analysis, we used a similar scheme suggested by Kim *et al* [5]. We validated the panel using reference materials (RM) with known VAF through analysis of the accuracy, sensitivity, specificity, and precision. For analysis of the limit of detection, we performed a droplet digital PCR assay to accurately discern the VAF of diluted RMs.

For hereditary cancer analysis, the sequence reads were processed for base calling, demultiplexing, and alignment to the hg19 human reference genome (GRCh37/hg19), and SNVs or INDELs were called using the Genome Analysis Toolkit HaplotypeCaller. Copy-number variants (CNVs) were called using the read-depth ratio of the observed and predicted read depth[6]. All variants were classified into pathogenic, likely pathogenic, uncertain significance, likely benign, or benign, following the 2015 American College of Medical Genetics and Genomics (ACMG) standards and guideline[7].

Statistical analysis

All statistical analyses were performed using R 3.5.1. The *P* value was calculated from the Fisher's exact test for the comparison of values between groups. *P* values less than 0.05 were considered to indicate significant differences.

References

1. Li H, Durbin R. Fast and accurate long-read alignment with Burrows-Wheeler transform. Bioinformatics. 2010;26(5):589-95.

2. McKenna A, Hanna M, Banks E, Sivachenko A, Cibulskis K, Kernytsky A, et al. The Genome

Analysis Toolkit: a MapReduce framework for analyzing next-generation DNA sequencing data. Genome Res. 2010;20(9):1297-303.

3. Cibulskis K, Lawrence MS, Carter SL, Sivachenko A, Jaffe D, Sougnez C, et al. Sensitive detection of somatic point mutations in impure and heterogeneous cancer samples. Nat Biotechnol. 2013;31(3):213-9.

4. Lek M, Karczewski KJ, Minikel EV, Samocha KE, Banks E, Fennell T, et al. Analysis of protein-coding genetic variation in 60,706 humans. Nature. 2016;536(7616):285-91.

5. Min YK, Lee YK, Nam SH, Kim JK, Park KS, Kim JW. Quantitative and Qualitative QC of Next-Generation Sequencing for Detecting Somatic Variants: An Example of Detecting Clonal Hematopoiesis of Indeterminate Potential. Clin Chem. 2020;66(6):832-41.

6. Kang Y, Nam SH, Park KS, Kim Y, Kim JW, Lee E, et al. DeviCNV: detection and visualization of exon-level copy number variants in targeted next-generation sequencing data. BMC Bioinformatics. 2018;19(1):381.

7. Richards S, Aziz N, Bale S, Bick D, Das S, Gastier-Foster J, et al. Standards and guidelines for the interpretation of sequence variants: a joint consensus recommendation of the American College of Medical Genetics and Genomics and the Association for Molecular Pathology. Genet Med. 2015;17(5):405-24.
